# The effect of statins on prostate cancer recurrence and mortality after definitive therapy: a systematic review and meta-analysis

**DOI:** 10.1038/srep29106

**Published:** 2016-07-07

**Authors:** Ping Tan, Shiyou Wei, Lu Yang, Zhuang Tang, Dehong Cao, Liangren Liu, Junhao Lei, Yu Fan, Liang Gao, Qiang Wei

**Affiliations:** 1 Department of Urology, West China Hospital, Sichuan University, Chengdu, Sichuan, People’s Republic of China; 2 Institute of Urology, West China Hospital, Sichuan University, Chengdu, Sichuan, People’s Republic of China; 3 Department of Cardiovascular and Thoracic Surgery, West China Hospital, Sichuan University, Chengdu, Sichuan, People’s Republic of China

## Abstract

In this work, we aim to further analyze the association of statins use with biochemical recurrence (BCR) of prostate cancer (PCa) and PCa-specific mortality after definitive therapy. A systematic literature search of PubMed, MEDLINE, and EMBASE through Jul 2015 was conducted. Pooled Hazard ratio (HR) estimates with corresponding 95% confidence intervals (CIs) were calculated using random-effects model. STATA version 10 (Stata corporation, college station, TX) was employed to conduct all statistical analyses. A total of 22 and 8 studies contributed to the biochemical recurrence analysis and PCa-specific mortality, respectively. 13 trials were included for BCR-free survival analysis. The combined result showed statins users had lowered 12% BCR risk of PCa compared with non-users (HR = 0.88, 95%CI: 0.765–0.998) (p < 0.05). The association was null among the men who underwent radical prostatectomy as primary therapy (HR = 0.96, 95%CI: 0.83–1.09), while the improved outcomes had be seen among patients who received radiation therapy (HR = 0.67, 95%CI: 0.48–0.86). After excluding the patients undergoing ADT, participants did not benefit from statins use (HR = 0.94, 95%CI: 0.77–1.11). Meanwhile, long-term statins using did not alter recurrence risk. A lower risk of prostate cancer-specific mortality was observed among statins users (HR = 0.68, 95%CI: 0.56–0.80). There was a plausible trend towards increasing the BCR-free survival rate among statins users.

Prostate cancer (PCa) is the most commonly diagnosed malignancy among men in the US. Approximately 220,800 American males are estimated to have been diagnosed with prostate cancer in 2015, and 27,540 will die of the disease^1^. Unfortunately, up to 40% of patients experienced postoperative disease recurrence or progression on long-term follow-up^2^. Statins are wildly used medications for lowering hypercholesterolemia. Interestingly, some reports declaims that statins have been associated with a reduced incidence of PCa, with evidence being particularly strong for advanced PCa^3^,^4^. This effect may be related to both cholesterol and non-cholesterol-mediated mechanisms. While, the impact of statins use on the incidence and natural history of PCa remains controversial^5^,^6^. Moreover, there is increasingly considerable interest in the potential ability of statins to improve PCa outcomes and decrease recurrence risk after definitive therapy.

A recent meta-analysis, including a total of 13 studies, provided the evidence that statins use could not reduce the recurrence risk among men treated with definitive therapy^7^. While, these studies included in Park *et al*.^7^ covered heterogeneous and limited sized cohorts. Moreover, nine more studies were published after that^7^ and provided further evidence on this topic. Thus, a further analysis of the association of statins use with recurrence risk after definitive therapy for PCa is warrant. Furthermore, whether statins have effect on the PCa-specific mortality still remained unknown.

## Methods

### Study selection

We performed a literature search without language restrictions using the databases of PubMed (Jan 1967–April 2015), MEDLINE (Jan 1967–April 2015), EMBASE (Jan 1990–April 2015) to include all studies that investigate the association of statins use with prostate cancer outcomes. In addition, a manual search was conducted to identify additional relevant studies. After removing duplicate publications, two reviewers (Tan & Wei) independently assessed all remaining results by checking titles and abstracts. All publications, including abstracts, were eligible for retrieval. When studies reported outcomes from similar or overlapping databases or cohorts, only data from the most recent publication were included. The studies included should analyze the BCR, PCa-specific mortality, or BCR-free survival. BCR defined as a post-treatment PSA value of 0.2 ng/ml or greater in men who underwent radical prostatectomy; nadir PSA level +2 ng/ml (Phoenix criteria), for men treated with radiation therapy; or any PSA increase in men treated with primary androgen deprivation therapy; and not any evidence of clinical and/or radiographically detected disease. We adapted a PRISMA (preferred reporting items for systematic reviews and meta-analyses) flow-chart to depict the study selection.

### Data extraction and analysis

Data from each study were independently extracted by two reviewers (Tan & Wei) using a standardized data-extraction form. Any disagreements were resolved by consensus or by consultation with a third reviewer (Yang). Adjusted multivariate Hazard ratio (HR) with corresponding 95% confidence intervals (CIs) were used to assess potential association between statins use and BCR and mortality of prostate cancer following treatment. In addition, we also tried to contact authors via e-mail to obtain further information that had not been reported in their published articles. We identified heterogeneity between studies using the standard Cochran’s Q test with a significance level of α = 0.10. We also examined heterogeneity with the *I^2^* statistic, which quantifies inconsistency across studies to assess the impact of meta-analysis heterogeneity. An *I^2^* statistic of 50% or more indicates a considerable level of heterogeneity. When heterogeneity was found, we attempted to determine potential sources of heterogeneity through stratification by various factors, and inference analysis and exclusion sensitivity analyses. Publication bias was detected using both the Begg’s and Egger’s tests. Statistical significance was determined using the two-tailed test, where *P* < 0.05 was considered significant. STATA version 10 (Stata corporation, college station, TX) was employed to conduct all statistical analyses.

## Results

A total of 3229 publications were identified during the initial search (see Additional file 1.), and after employing exclusion criteria, a total of 22 studies were included for PCa recurrence analysis^8^,^9^,^10^,^11^,^12^,^13^,^14^,^15^,^16^,^17^,^18^,^19^,^20^,^21^,^22^,^23^,^24^,^25^,^26^,^27^,^28^,^29^ (Table 1), 8 trials were available for PCa-specific mortality^22^,^30^,^31^,^32^,^33^,^34^,^35^,^36^ (Table 2), and 13 for BCR-free survival analysis^8^,^9^,^11^,^14^,^17^,^20^,^25^,^29^,^36^,^37^,^38^,^39^,^40^ (Table 3). Among 22 studies included in meta-analysis for PCa recurrence, 14 included a population primarily treated with radical prostatectomy (RP). Meanwhile, men in seven studies were treated with radiation therapy (RT-either external beam, brachytherapy or a combination of them). And other one study in which included patients were treated with RP, RT with or without ADT (androgen deprivation therapy) or ADT only^22^. Among 8 trials included for PCa specific mortality, one included a population of diabetic men^34^. Two abstract were included in BCR-free survival analysis.

### Statins and biochemical recurrence risk of PCa

**Figure 1:**
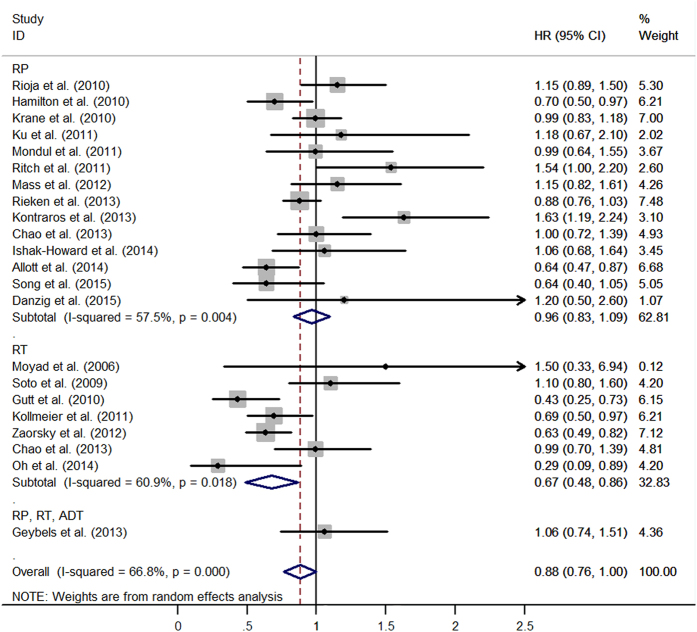
The effect of statins on BCR risk of prostate cancer among men following definitive therapy.

**Figure 2:**
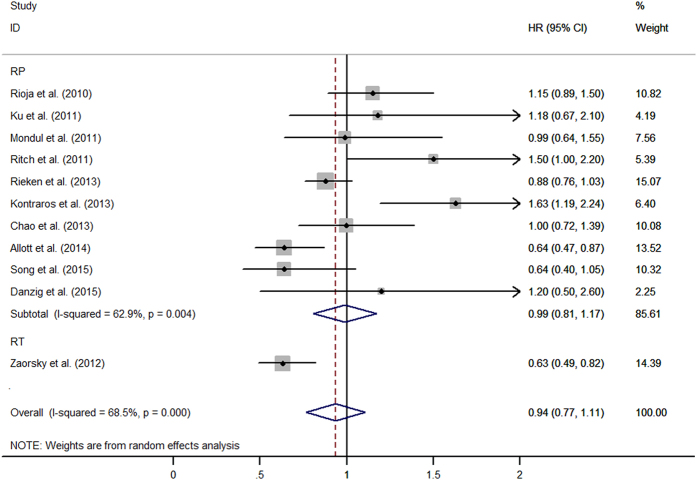
The effect of statins on BCR risk of prostate cancer among men who did not receive ADT pre- or post-local therapy.

Most studies demonstrated that statins had a neutral effect on recurrence after prostate cancer diagnosis. Overall, the combined result showed statins users had lowered the risk of recurrence compared with non-users (HR = 0.88, 95%CI: 0.765–0.998, *I*^*2*^ = 65.6%, *p* < 0.001) (Fig. 1). The association was null among the men who underwent RP as primary therapy (HR = 0.96, 95%CI: 0.83–1.09, *I*^*2*^ = 57.5%, *p* = 0.004). However, the combined result showed an inverse association between statins use and recurrence after treatment of RT (HR = 0.67, 95%CI: 0.48–0.86, *I*^*2*^ = 60.9%, *p* < 0.001). It is really important to exclude patients undergoing ADT, in order to reduce the correlation between metabolic syndrome secondary androgen deprivation and BCR, as reported in literature. After excluding patients undergoing ADT, 11 studies were included and the combined estimates suggested that no patient had benefit from statins use after local therapy (HR = 0.94, 95%CI: 0.77–1.11) (Fig. 2). Additional file 2 and file 3 shown, long-term statins use still cannot alter recurrence risk (HR = 0.90, 95%CI: 0.72–1.07). No publication bias was detected among 22 studies when Begg’s and Egger’s tests were conducted (*p* = 0.81, *p* = 0.87, respectively) (see Additional file 4).

In the stratified analysis, the pooled HR estimates suggested that reverse relationship between statins use and PCa recurrence was observed in the US (HR = 0.86, 95%CI: 0.73–0.99). However, we failed to find a similar relation in the non-US (HR = 1.01, 95%CI: 0.67–1.35). When stratified analyses were performed on the results with or without adjusted for age, BMI, or PSA, we observed that statins use had a neutral effect on PCa recurrence when adjusted for them. Post-operation and pre-operation statins use both cannot alter PCa recurrence risk. Detailed data are illustrated in Table 4.

While there was limited data of the effect of statins on local recurrence and metastasis of PCa after treatment. For low-, intermediate- and high-risk group, statins use did not affect the recurrence risk^14^. The Mondual *et al*. also observed that statins using was not associated with the progression to metastasis or death of PCa after treatment^16^.

### Statins and prostate cancer specific mortality

**Figure 3:**
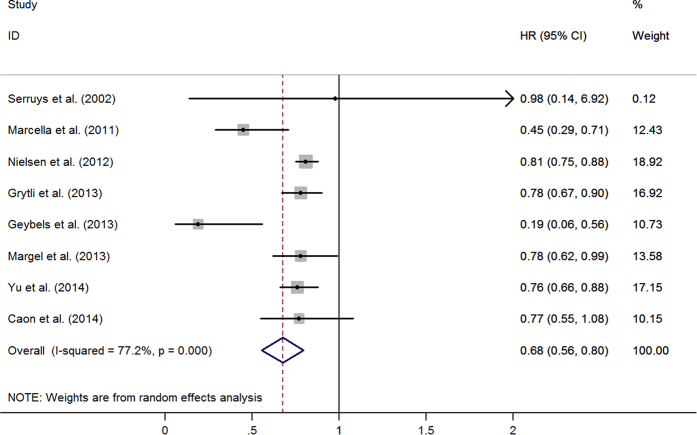
The relationship between statins use and prostate cancer-specific mortality.

Eight studies evaluated exposure to statins and the recurrence of PCa. Figure 3 graphed the HRs and 95%CIs from individual studies and the pooled results. Among them, six studies showed an inverse association. The combined estimates of eight studies showed a decreased risk (HR = 0.68, 95%CI: 0.56–0.80) (Table 5). After excluding Serruys *et al*.^30^, due to its large confidence interval (HR = 0.98, 95%CI: 0.14–6.92), the result of seven studies was consistent and stable (HR = 0.68, 95%CI: 0.55–0.80). There was no publication bias (Begg’s test, *p* = 0.386; Egger’s test, *p* = 0.063). After adjusting for age, BMI, or PSA, the same trends were observed in these subgroups (see Table 5).

### Effect of statins on BCR-free survival

Table 3 showed 13 retrospective studies were included to evaluate the effect of statins on biochemical recurrence-free survival. The 5-year BCR-free survival rate of statins users ranged from 63.7 to 97.2% and that was 57–89.6% for non-users. The statins users had much higher 10-year BCR-free survival rate compared with non-users.

### Sensitivity analysis

Sensitivity analyses were conducted to evaluate the effect of each study on the overall estimates by sequentially excluding each study in turn. In our meta-analysis, we found that probably no study could affect the summary of risk estimates, means that the results remained stable (data not shown).

## Discussion

In this meta-analysis among men following definitive treatment of localized prostate cancer, approximately 12% reduction risk of recurrence in statins users was found (p < 0.001). In cumulative meta-analysis, a reduction risk was found for the first time in 2010, when Hamilton *et al*.^12^ joined the analysis. When analyzed by treatment modality, statins use had a neutral effect on BCR risk among men who underwent radical prostatectomy. On the contrary, there was almost 33% lower risk of recurrence among statins users who received RT as primary treatment modality. These are consistent with the results from subgroup patients who did not receive adjuvant ADT. The benefits were null when estimates controlled for age, BMI or PSA. Overall, there was 32% lower risk of prostate cancer-specific mortality among statins users. To our knowledge, this is the first meta-analysis to address the relationship between PCa-specific mortality and statins use.

Whether the improved outcomes seen among patients who underwent RT reflecting that statins directly inhibit PCa progression, act as a radiosensitizer, or are the result of either residual confounding or type I error of studies is unknown. One postulated possible explanation was statin-induced radiosensitizing effects. Prostate cancer cell death was increased in both *in vitro* and *in vivo* models when combined the statins and ionizing radiation, which might attributed to the MYC oncogene, as statins decreasing cellular MYC levels^11^,^41^. Statins also play a synergistic role with radiation by promoting autophagy pathway, causing prostate cancer cell death^42^. Moreover, Gutt *et al*.^11^ proved that a larger benefit was seen in men treated with lower radiation doses. Clinically, RT combined with ADT were often used to treat PCa. In our work, seven studies analyzed men who treated by RT, but six of them included patients who received ADT. As ADT could reduce PCa recurrence risk, the effect of statins could not be confirmed there.

Intriguingly, our findings do not support the hypothesis that statins, when taken at low dose for managing hypercholesterolemia, can reduce the risk of PCa recurrence among men treated with RP as primary therapy. Although many studies found the statins had antineoplastic properties, two precious high quality meta-analyses had confirmed that statins use could not reduce the risk of prostate cancer^5^,^43^,^44^. Another plausible explanation is that statins are associated with a dose-dependent reduction in the risk of biochemical recurrence. The effect of statins on lowering recurrence risk was observed only when taken at doses ≥1 DE (dose equivalents)^12^. Many *in vitro* studies that demonstrated these protective effects also used statins concentrations that were much higher than those seen with standard therapeutic use.

This meta-analysis suggested that regular statins use reduced 32% risk of PCa-related mortality. Meanwhile, Marcella *et al*.^31^ evaluated effect of statin type on prostate cancer mortality, which showed that hydrophilic and lipophilic statins both lowered the risk of PCa-specific mortality. However, there was a trend based on potency in which high-potency statins were associated with a 73% risk reduction, whereas this effect was not observed in low-potency statins.

One of 13 trials evaluating the BCR-free survival suggested that statins users had a lower 5-year BCR-free survival rate compared with non-users^17^, while some reveled reversely^9^,^25^,^37^ or showed there was no difference between two groups^20^. Meanwhile, the BCR-free survival rate at 8 or 9-year were still inconsistent^8^,^14^,^40^. However, the BCR-free survival rate at 10 year among statins users was much higher compared with non-users^36^,^38^. These inconsistencies might be attributed to the composition of risk group of patients, as lower-risk group had higher 5-year BCR-free survival rate compared with intermediate-risk and high-risk group^9^,^11^,^14^,^17^. Meanwhile, treatment modality and the radiation doses among men who underwent RT also contributed to this inconsistency^12^. Overall, there is a plausible trend towards increasing the BCR-free survival rate among statins users.

Given the known that income, education and health insurance coverage influence access to appropriate early detection, statins users are more likely to get PSA testing done and treatment, leading percent of low or intermediate-risk patients much higher among statins users than that in non-users. This might cause an illusion that statins have protective effect. The possible confounding effect arising due to the indications for which statins are prescribed also needs to be emphasized. Statins users are more likely to be obese or older as compared to non-users. This could affect PCa outcomes or progression. The subgroup analyses of 6 studies which adjusted for BMI or age reveled a neutral effect on BCR. This effect was also observed in the combined estimates of four studies adjusted for both BMI and age.

A previous meta-analysis, including 8 studies, initially addressed the relationship between statins use and PCa recurrence and showed that there was no association between statins use and recurrence after prostate cancer diagnosis (HR = 0.91, 95%CI: 0.72–1.13), this effect was consistent with men received RP or RT^45^. After that, another meta-analysis included 13 studies in the formal meta-analysis was conducted, which showed the same relationship (HR = 0.90, 95%CI: 0.74–1.08), while a reverse association was initially observed among men accepted RT (HR = 0.68, 95%CI: 0.49–0.93)^7^. Compared with Park *et al*.^7^, after including 9 more new studies that provided stronger evidence, we found a reverse association with statins among men following definitive treatment. Besides, Oh *et al*.^25^ updated their data in 2014, which may also contribute to this inconsistency. Unexpectedly, the combined result of these 10 studies showed a neutral effect of statins use on recurrence risk (HR = 0.88, 95%CI: 0.70–1.05).

Some limits in our meta-analysis should be mentioned. First, the available literature was limited to English language, which might be subject to selective bias and confounding. Second, there was significant heterogeneity among studies that might be attributed to various percent of risk groups. In a study, the beneficial effect was limited to high-risk patients, which proved that risk groups could affect the recurrence-free survival^14^. While, the data in different risk groups was limited; thus, the future studies addressing this topic should take the risk groups into account. Another potential factor contributed to this heterogeneity was starting time of statins use, which means some patients started using statins before operation (RT or RP or both), some started using during operation, while others received statins for the first time after operation. Third, the overall result showed that statins use lowered the recurrence of PCa, while the upper confidence interval close to 1.00. Therefore the result of this part needs to be explained with caution. At last, limited prospective sequential data was available at the moment, lowering the strength of evidence in this meta-analysis.

## Conclusion

In summary, our work demonstrated that statins use could not lower PCa recurrence risk among all patients underwent RP. Although the improved outcomes might be seen among statins users who underwent RT, more future studies excluded men who received ADT pre- or post-radiation therapy should be done. In addition, statins use decreased the prostate cancer-specific mortality risk. A plausible trend towards increasing the BCR-free survival rate among statins users was also observed.

## Additional Information

**How to cite this article**: Tan, P. *et al*. The effect of statins on prostate cancer recurrence and mortality after definitive therapy: a systematic review and meta-analysis. *Sci. Rep.* **6**, 29106; doi: 10.1038/srep29106 (2016).

## Supplementary Material

Supplementary Information

## Figures and Tables

**Table 1:** Study characteristics of 22 included studies of the association between statins use and biochemical recurrence risk of PCa after definitive therapy.

Study (year)	Country	Primary treatment(s)	No. of patients	No. of patients on statins	No. of recurrence patients	ADT (%)	Estimate(s)(95% confidence interval, CI)
Moyad *et al*. (2006)^8^	US	brachytherapy+EBRT	938	191	39	40.7	HR:1.5 (0.33–6.94)
Soto *et al*. (2009)^9^	US	3D-RT or IMRT	968	220	NR	28.9	HR:1.1 (0.8–1.6)
Rioja *et al*. (2010)^10^	US	RP	3748	2664	249	0.0	HR:1.15 (0.89–1.50)
Gutt *et al*. (2010)^11^	US	brachytherapy+EBRT	691	189	113	41.0	HR:0.43(0.25–0.73)
Hamilton *et al*. (2010)^12^	US	RP	1319	236	304	18.0	HR:0.70 (0.50–0.97)
Krane *et al*. (2010)^13^	US	rRP	3828	1031	NR	1.5	HR:0.99 (0.83–1.18)
Kollmeier *et al*. (2011)^14^	US	3D-RT or IMRT	1681	382	301	56.0	HR:0.69 (0.50–0.97)
Ku *et al*. (2011)^15^	Korean	RRP	687	87	145	0.0	HR:1.18 (0.67–2.10)
Mondul *et al*. (2011)^16^	US	RRP	2399	779	127	0.0	HR:0.99 (0.64–1.55)
Ritch *et al*. (2011)^17^	US	RP	1261	281	NR	0.0	HR:1.54 (1.00–2.20)
Zaorsky *et al*. (2012)^18^	US	3D-CRT+IMRT	2051	691	177	0.0	HR:0.63 (0.49–0.82)
Mass *et al*. (2012)^19^	US	ORRP	1446	437	166	NR	HR:1.15 (0.82–1.61)
Rieken *et al*. (2013)^20^	Multicenter	RP	6842	2275	778	0.0	HR:0.88 (0.76–1.03)
Kontraros *et al*. (2013)^21^	Greece	RP	588	107	187	0.0	HR:1.63 (1.19–2.24)
Geybels *et al*. (2013)^22^	US	RP, RT	1001	289	151	NR	HR:1.06 (0.74–1.54)
Chao *et al*. (2013)^24^	US	EBRT	774	401	145	67.0	HR:0.99 (0.70–1.39)
Chao *et al*. (2013)^24^	US	RP	1184	446	156	0.0	HR:1.00 (0.72–1.39)
Oh *et al*. (2014)^25^	US	Brachytherapy+EBRT	247	174	18	25.9	HR:0.29 (0.09–0.89)
ishak-Howard *et al*. (2014)^26^	US	RRP	539	258	115	NR	HR:1.06 (0.68–1.64)
Allott *et al*. (2014)^27^	US	RP	1146	400	402	0.0	HR:0.64 (0.47–0.87)
Danzig *et al*. (2015)^29^	US	RP	669	76	147	0.0	HR:1.20 (1.50–2.60)
Song *et al*. (2015)^28^	Korea	rRP or ORRP	1896	211	466	0.0	HR:0.64 (0.40–1.05)

Note: XRT, external beam radiotherapy (XRT); RRP, radical retropubic prostatectomy; 3D-CRT, 3-dimensional conformal radiation therapy; IMRT, intensity modulated radiation therapy; ORRP, open radical retropubic prostatectomy; rRP, robotic radical prostatectomy.

**Table 2:** Study characteristics of 8 studies of the association between statins use and prostate cancer-specific mortality.

Study (year)	Country	Duration of follow-up (Mean ± SD)	All male subjects (%)	No. of died from PCa*	Estimate(s)(95% confidence interval, CI)
Serruys *et al*. (2002)^30^	Multicenter	3.9y	1406 (83.8)	4	HR:0.98 (0.14–6.92)
Marcella *et al*. (2011)^31^	US	NR	760 (100.0)	380	HR:0.45 (0.29–0.71)
Nielsen *et al*. (2012)^32^	Denmark	2.6y	27752 (100.0)	10542	HR:0.81 (0.75–0.88)
Grytli *et al*. (2013)^33^	Norway	39 M	3699 (100.0)	NR	HR:0.78 (0.67–0.90)
Geybels *et al*. (2013)^22^	US	6.1y	1001 (100.0)	39	HR:0.19 (0.06–0.56)
Margel *et al*. (2013)^34^	Canada	4.64y	3837 (100.0)	291	HR:0.78 (0.62–0.99)
Yu *et al*. (2014)^35^	UK	4.4y ± 2.9	11772 (100.0)	1791	HR:0.76 (0.66–0.88)
Caon *et al*. (2014)^36^	Canada	8.4y	3851 (100.0)	NR	HR:0.77 (0.55–1.08)

^*^PCa, prostate cancer.

**Table 3:** Characteristics of 13 studies evaluating the effect of statins on Biochemical Recurrence-Free Survival.

Sources (year)	Primary treatment(s)	No. of patients	Biochemical Recurrence-Free Survival Rate
			Duration: Statins users (%) vs Non-statin users (%); *P*-value
Katz *et al*. (2003)^37^	RT#	905	5 year: 88.8% vs 71.2%; *P* = .009
Moyad *et al*. (2006)^8^	RT	938	9 year: 98.4% vs 95.2%; *P* = 0.062
Shippy *et al*. (2007)^38^	RT	871	10-year: 76% vs 66%; *P* = 0.01
Soto *et al*. (2009)^9^	RT	968	5 year: 67% vs 57%; *P* = 0.03
Gutt *et al*. (2010)^11^	RT	691	4 year: 93% vs 80%; *P* < 0.001
Kollmeier *et al*. (2011)^14^	RT	1711	5 year: 89% (95%CI, 85–92%) vs 80% (95%CI, 72–86%) 8 year: 83% (95%CI, 81–85%) vs 74% (95%CI, 71–77%)
Ritch *et al*. (2011)^17^	RP$	1261	5 year: 75% vs 84%; *P* < 0.05
Misrai *et al*. (2012)^39^	RP	377	2 year: 93% vs 88%; *P* = 0.16
Rieken *et al*. (2013)^20^	RP	6842	2 year: 94 ± 1% vs 92 ± 0%; *P* = NR* 5 year: 84 ± 1% vs 82 ± 1%; *P* = NR 10 year: 71 ± 3% vs 66 ± 3%; *P* = NR
Caon *et al*. (2014)^36^	RT ± ADT	3851	10-year: 94.1% vs 91.2%; *P* = 0.031
Oh *et al*. (2014)^25^	RT	247	5 year: 97.2% vs 89.6%; *P* = 0.007
Cuaron *et al*. (2015)^40^	RT	754	8-year: 84.5% vs 88.2%; *P* = 0.85
Danzig *et al*. (2015)^29^	RP	767	2-year: 79.0% vs 79.3%; *P* = NR 5-year: 63.7% vs 72.4%; *P* = NR

^*^NR, not report.

^#^RT, radiation therapy.

^$^RP, radical prostatectomy.

**Table 4:** Pooled estimates of BCR analyses in subgroups.

Sources	No. of studies	Pooled estimates		Tests of heterogeneity		Tests of publication bias	
		HR	95%CI	*I*^*2*^(%)	*p-*val.	Begg’s *p*-val.	Egger’s *p*-val.
All studies	22	0.88	0.765–0.998	66.8	<0.001	0.809	0.866
Treatment modality							
RP	14	0.96	0.83–1.09	57.5	0.004	0.784	0.518
RT	7	0.67	0.48–0.86	60.9	0.018	0.881	0.753
Exclude pts received ADT	11	0.94	0.77–1.11	68.5	<0.001	0.533	0.493
RP	10	0.99	0.81–1.17	62.9	0.004	0.788	0.500
RT	1	0.63	0.63–0.79	–	–	–	–
Include pts received ADT	8	0.74	0.54–0.95	71.5	0.001	0.712	0.453
RT	6	0.69	0.43–0.96	67.5	0.009	0.851	0.495
RP	2	0.86	0.57–1.14	73.4	0.052	–	–
Pre- or post-operation	7	0.87	0.69–1.04	51.9	0.052	1.000	0.760
Post-operation	5	0.83	0.61–1.05	59.0	0.045	0.806	0.740
Pre-operation	2	1.00	0.75–1.24	0.0	0.967	–	–
Results for long-term statins use	7	0.90	0.72–1.07	11.8	0.340	0.548	0.529
Park *et al*. analysis							
Before	12	0.89	0.72–1.06	70.5	<0.001	0.945	0.994
After	10	0.88	0.70–1.05	65.0	0.002	0.929	0.865
Country							
US	18	0.86	0.73–0.99	66.6	<0.001	0.879	0.710
Non-US	4	1.01	0.67–1.35	71.6	0.014	0.497	0.730
PSA							
Adjusted	11	0.95	0.81–1.09	52.5	0.021	0.697	0.401
Not adjusted	11	0.80	0.60–0.99	73.3	<0.001	0.697	0.592
BMI							
Adjusted	6	0.93	0.71–1.16	71.9	0.003	0.851	0.943
Not adjusted	16	0.86	0.72–1.01	66.5	<0.001	0.928	0.948
Age							
Adjusted	8	0.96	0.81–1.12	59.0	0.017	0.621	0.495
Not adjusted	14	0.82	0.65–0.98	65.5	<0.001	0.913	0.873
BMI & Age							
Adjusted	4	1.00	0.68–1.32	80.1	0.002	0.497	0.954
Not adjusted	18	0.86	0.73–0.98	63.5	<0.001	1.000	0.967
BMI or PSA or Age							
Adjusted	12	0.95	0.83–1.07	50.3	0.023	0.681	0.490
Not adjusted	10	0.76	0.55–0.97	68.2	0.001	0.929	0.865

Note: HR, Hazard ratio; 95%CI, 95% confidence intervals; PCa, prostate cancer; PSA, prostate-specific antigen; RT, radiation therapy; RP, radical prostatectomy. BCR, biochemical recurrence.

**Table 5:** Pooled estimates of Mortality analyses in subgroups.

Sources	No. of studies	Pooled estimates		Tests of heterogeneity		Tests of publication bias	
		HR	95%CI	*I*^*2*^(%)	*p-*val.	Begg’s *p*-val.	Egger’s *p*-val.
PCa-specific mortality	8	0.68	0.56–0.80	77.2	<0.001	0.386	0.063
Exclude Serruys *et al*.	7	0.68	0.55–0.80	80.5	<0.001	0.072	0.018
Age							
Adjusted	5	0.64	0.48–0.79	87.0	<0.001	0.086	0.004
Not adjusted	2	0.78	0.60–0.97	0.0	0.908	–	–
BMI							
Adjusted	2	0.62	0.32–0.92	84.8	0.010	–	–
Not adjusted	5	0.68	0.50–0.85	82.0	<0.001	0.806	0.258
PSA							
Adjusted	3	0.61	0.35–0.86	89.4	<0.001	1.000	0.077
Not adjusted	4	0.70	0.51–0.89	70.9	0.016	0.734	0.445
BMI & Age							
Adjusted	2	0.62	0.32–0.92	84.8	0.010	–	–
Not adjusted	5	0.68	0.50–0.85	82.0	<0.001	0.806	0.258
BMI or PSA or Age							
Adjusted	5	0.64	0.48–0.79	87.0	<0.001	0.086	0.004
Not adjusted	2	0.78	0.60–0.97	0.0	0.908	–	–

Note: HR, Hazard ratio; 95%CI, 95% confidence intervals; PCa, prostate cancer; PSA, prostate-specific antigen.

## References

[b1] National Cancer Institute (2015) SEER stat fact sheets; Prostate. Available: http://seer.cancer.gov/statfacts/html/prost.html. Accessed April 2015.

[b2] Boorjian S. A. . A critical analysis of the long-term impact of radical prostatectomy on cancer control and function outcomes. Eur Urol 61, 664–675, 10.1016/j.eururo.2011.11.053 (2012). 22169079

[b3] Lustman A., Nakar S., Cohen A. D. & Vinker S. Statin use and incident prostate cancer risk: does the statin brand matter? A population-based cohort study. Prostate Cancer P. D. 17, 6–9, 10.1038/pcan.2013.34 (2013). 24061633

[b4] Lehman D. M., Lorenzo C., Hernandez J. & Wang C. P. Statin use as a moderator of metformin effect on risk for prostate cancer among type 2 diabetic patients. Diabetes Care 35, 1002–1007, 10.2337/dc11-1829 (2012). 22456867 PMC3329836

[b5] Bonovas S., Filioussi K. & Sitaras N. M. Statin use and the risk of prostate cancer: A metaanalysis of 6 randomized clinical trials and 13 observational studies. Int J Cancer 123, 899–904, 10.1002/ijc.23550 (2008). 18491405

[b6] Bansal D., Undela K., D’Cruz S. & Schifano F. Statin use and risk of prostate cancer: a meta-analysis of observational studies. PLoS One 7, e46691, 10.1371/journal.pone.0046691 (2012). 23049713 PMC3462187

[b7] Park H. S. . Statins and prostate cancer recurrence following radical prostatectomy or radiotherapy: a systematic review and meta-analysis. Ann Oncol 24, 1427–1434, 10.1093/annonc/mdt077 (2013). 23508824 PMC3660083

[b8] Moyad M. A. . Statins, especially atorvastatin, may improve survival following brachytherapy for clinically localized prostate cancer. Urol Nurs 26, 298–303 (2006). 16939047

[b9] Soto D. E., Daignault S., Sandler H. M. & Ray M. E. No effect of statins on biochemical outcomes after radiotherapy for localized prostate cancer. Urology 73, 158–162, 10.1016/j.urology.2008.02.055 (2009). 18722651

[b10] Rioja J. . 125 Impact of statin use on pathologic features in men treated with radical prostatectomy. J. Urology 183, e51 (2010).

[b11] Gutt R. . Statin use and risk of prostate cancer recurrence in men treated with radiation therapy. J Clin Oncol 28, 2653–2659, 10.1200/jco.2009.27.3003 (2010). 20421534

[b12] Hamilton R. J. . Statin medication use and the risk of biochemical recurrence after radical prostatectomy: results from the Shared Equal Access Regional Cancer Hospital (SEARCH) Database. Cancer 116, 3389–3398, 10.1002/cncr.25308 (2010). 20586112 PMC3188453

[b13] Krane L. S. . Men presenting for radical prostatectomy on preoperative statin therapy have reduced serum prostate specific antigen. J Urol 183, 118–124, 10.1016/j.juro.2009.08.151 (2010). 19913252

[b14] Kollmeier M. A. . Improved biochemical outcomes with statin use in patients with high-risk localized prostate cancer treated with radiotherapy. Int J Radiat Oncol Biol Phys 79, 713–718, 10.1016/j.ijrobp.2009.12.006 (2011). 20452139

[b15] Ku J. H. . Relationship of statins to clinical presentation and biochemical outcomes after radical prostatectomy in Korean patients. Prostate Cancer P. D. 14, 63–68, 10.1038/pcan.2010.39 (2011). 20938462

[b16] Mondul A. M. . Association of statin use with pathological tumor characteristics and prostate cancer recurrence after surgery. J Urol 185, 1268–1273, 10.1016/j.juro.2010.11.089 (2011). 21334020 PMC3584560

[b17] Ritch C. R., Hruby G., Badani K. K., Benson M. C. & McKiernan J. M. Effect of statin use on biochemical outcome following radical prostatectomy. BJU Int 108, E211–216, 10.1111/j.1464-410X.2011.10159.x (2011). 21453350

[b18] Zaorsky N. G., Buyyounouski M. K., Li T. & Horwitz E. M. Aspirin and statin nonuse associated with early biochemical failure after prostate radiation therapy. Int J Radiat Oncol Biol Phys 84, e13–17, 10.1016/j.ijrobp.2012.02.050 (2012). 22652109 PMC3423546

[b19] Mass A. Y., Agalliu I., Laze J. & Lepor H. Preoperative statin therapy is not associated with biochemical recurrence after radical prostatectomy: our experience and meta-analysis. J Urol 188, 786–791, 10.1016/j.juro.2012.05.011 (2012). 22818136

[b20] Rieken M. . Impact of statin use on biochemical recurrence in patients treated with radical prostatectomy. Prostate Cancer P. D. 16, 367–371, 10.1038/pcan.2013.31 (2013). 23999669

[b21] Kontraros M., Varkarakis I., Ntoumas K. & Deliveliotis C. Pathological characteristics, biochemical recurrence and functional outcome in radical prostatectomy patients on statin therapy. Urol Int 90, 263–269, 10.1159/000346751 (2013). 23548958

[b22] Geybels M. S. . Statin use in relation to prostate cancer outcomes in a population-based patient cohort study. Prostate 73, 1214–1222, 10.1002/pros.22671 (2013). 23633265 PMC3967507

[b23] Chao C. . Statin therapy is not associated with prostate cancer recurrence among patients who underwent radiation therapy. Cancer Lett 335, 214–218, 10.1016/j.canlet.2013.02.017 (2013). 23419526

[b24] Chao C. . Use of statins and prostate cancer recurrence among patients treated with radical prostatectomy. BJU Int 111, 954–962, 10.1111/j.1464-410X.2012.11639.x (2013). 23464862

[b25] Oh D. S. . Statin use is associated with decreased prostate cancer recurrence in men treated with brachytherapy. World J Urol 33, 93–97, 10.1007/s00345-014-1281-x (2014). 24671610

[b26] Ishak-Howard M. B., Okoth L. A. & Cooney K. A. Statin use and the risk of recurrence after radical prostatectomy in a cohort of men with inherited and/or early-onset forms of prostate cancer. Urology 83, 1356–1361, 10.1016/j.urology.2014.02.015 (2014). 24745796 PMC4230295

[b27] Allott E. H. . Postoperative statin use and risk of biochemical recurrence following radical prostatectomy: results from the Shared Equal Access Regional Cancer Hospital (SEARCH) database. BJU Int 114, 661–666, 10.1111/bju.12720 (2014). 24588774 PMC4153797

[b28] Song C. . Statin use after radical prostatectomy reduces biochemical recurrence in men with prostate cancer. Prostate 75, 211–217, 10.1002/pros.22907 (2015). 25327522

[b29] Danzig M. R. . Synergism between metformin and statins in modifying the risk of biochemical recurrence following radical prostatectomy in men with diabetes. Prostate Cancer P. D. 18, 63–68, 10.1038/cddis.2014.50010.1038/pcan.2014.47 (2015). 25403419

[b30] Serruys P. W. . Fluvastatin for prevention of cardiac events following successful first percutaneous coronary intervention: a randomized controlled trial. Jama 287, 3215–3222 (2002). 12076217 10.1001/jama.287.24.3215

[b31] Marcella S. W., David A., Ohman-Strickland P. A., Carson J. & Rhoads G. G. Statin use and fatal prostate cancer: a matched case-control study. Cancer 118, 4046–4052, 10.1002/cncr.26720 (2012). 22180145 PMC4314096

[b32] Nielsen S. F., Nordestgaard B. G. & Bojesen S. E. Statin use and reduced cancer-related mortality. N Engl J Med 368, 576–577, 10.1056/NEJMc1214827 (2013). 23388012

[b33] Grytli H. H., Fagerland M. W., Fossa S. D. & Tasken K. A. Association between use of beta-blockers and prostate cancer-specific survival: a cohort study of 3561 prostate cancer patients with high-risk or metastatic disease. Eur Urol 65, 635–641, 10.1016/j.eururo.2013.01.007 (2014). 23351721

[b34] Margel D. . Metformin use and all-cause and prostate cancer-specific mortality among men with diabetes. J Clin Oncol 31, 3069–3075, 10.1200/jco.2012.46.7043 (2013). 23918942

[b35] Yu O. . Use of statins and the risk of death in patients with prostate cancer. J Clin Oncol 32, 5–11, 10.1200/jco.2013.49.4757 (2014). 24190110

[b36] Caon J., Paquette M., Hamm J. & Pickles T. Does Statin or ASA Affect Survival When Prostate Cancer Is Treated with External Beam Radiation Therapy? Prostate Cancer 2014, 184297, 10.1155/2014/184297 (2014). 24729876 PMC3960556

[b37] Katz M. . Statin use is associated with improved biochemical outcome after high-dose radiotherapy for clinically localized prostate cancer. Int. J. Radiat. Oncol. 57, S271 (2003).

[b38] Shippy A. M., Katz M. S., Yamada Y. & Feder D. J. & MJ, Z. Statin use and clinical outcomes after high dose radiotherapy for prostate cancer “abstract”. Int. J. Radiat. Biol. 69, S113. Abstract 203. (2007).

[b39] Misrai V. . [Is statin use associated with D’Amico risk groups and biochemical recurrence after radical prostatectomy?]. Prog Urol 22, 273–278, 10.1016/j.purol.2011.11.001 (2012). 22515923

[b40] Cuaron J. . Statin use not associated with improved outcomes in patients treated with brachytherapy for prostate cancer. Brachytherapy 14, 179–184, 10.1016/j.brachy.2014.05.019 (2015). 25500364

[b41] Salih T. . In J Clin Oncol. (AMER SOC Clinical Oncology 2318 Mill Road, STE 800, Alexandria, VA 22314 USA).

[b42] He Z. . Cell killing and radiosensitizing effects of atorvastatin in PC3 prostate cancer cells. J Radiat Res (Tokyo) 53, 225–233 (2012). 22510595 10.1269/jrr.11114

[b43] Bonovas S., Filioussi K., Tsavaris N. & Sitaras N. M. Statins and cancer risk: a literature-based meta-analysis and meta-regression analysis of 35 randomized controlled trials. J Clin Oncol 24, 4808–4817, 10.1200/jco.2006.06.3560 (2006). 17001070

[b44] Dale K. M., Coleman C. I., Henyan N. N., Kluger J. & White C. M. Statins and cancer risk: a meta-analysis. Jama 295, 74–80, 10.1001/jama.295.1.74 (2006). 16391219

[b45] Scosyrev E. . Statin use and the risk of biochemical recurrence of prostate cancer after definitive local therapy: a meta-analysis of eight cohort studies. BJU Int 111, E71–77, 10.1111/j.1464-410X.2012.11527.x (2013). 23017100

