# Supplementary material for: The effect of statins on prostate cancer recurrence and mortality after definitive therapy: a systematic review and meta-analysis
Source: Sci Rep. 2016 Jul 7;6:29106. doi: 10.1038/srep29106 (PMC4935858; doi:10.1038/srep29106)

Additional Files

The effect of statins on prostate cancer recurrence and mortality after definitive therapy: a systematic review and meta-analysis

Ping Tan, M.D. [a, b,*], Shiyou Wei, M.D. [c,*], Lu Yang, Ph.D [a, b,*], Zhuang Tang, M.D. [a, b], Dehong Cao, M.D. [a, b], Liangren Liu, Ph.D [a, b], Junhao Lei, M.D. [a, b], Yu Fan, M.D. [a, b], Liang Gao, M.D. [a, b], Qiang Wei, M.D [a, b]

Additional file 1.Trial Identification, Inclusion, and Exclusion.

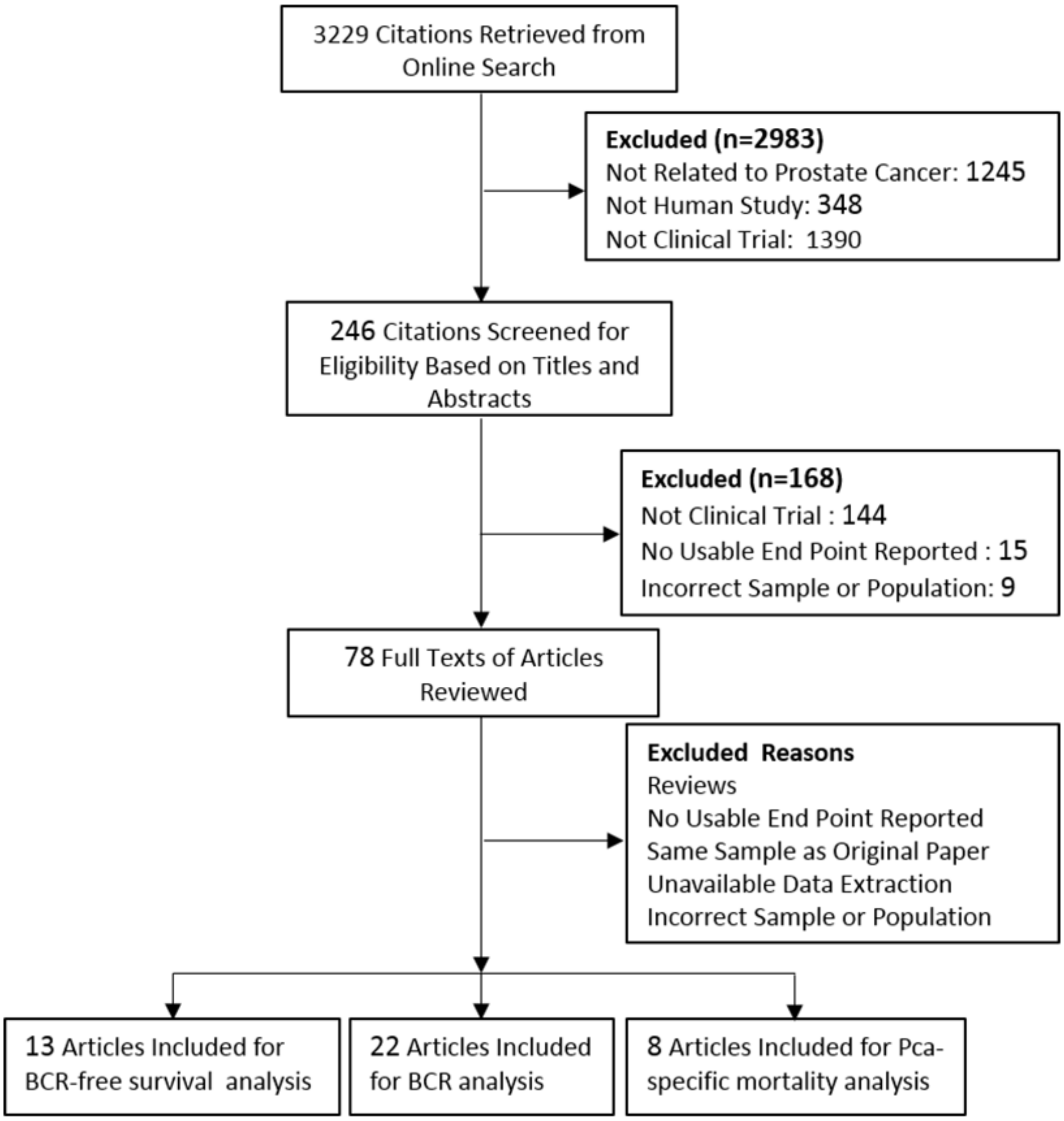

Additional file 2. Studies evaluating the association between long-term statins use and BCR risk of prostate cancer.

Study	HR#	95%CI	Recurrence cases	Definition of long-term statin use
Song et al. 2015 (Korea)	0.64	0.40-1.05	NR*	>36 months
ishak-Howard et al.2014(US)	0.932	0.53-1.63	NR	>5 years
Kontraros et al.2013(Greece)	1.63	1.02-2.60	NR	>2 years
Geybels et al.2013(US)	1.06	0.67-1.67	22	>5 years
Mondul et al.2011(US)	0.77	0.41-1.42	NR	>1 years
Chao et al.2013(US)	0.96	0.64-1.46	NR	>500 days
Chao et al.2013(US)	1.04	0.67-1.64	NR	>500 days

*NR, not report; #HR, Hazard Ratio; 95%CI, 95% confidence intervals.

Additional file 3. The meta-analysis of effect of long-term statins use on BCR risk of prostate cancer.

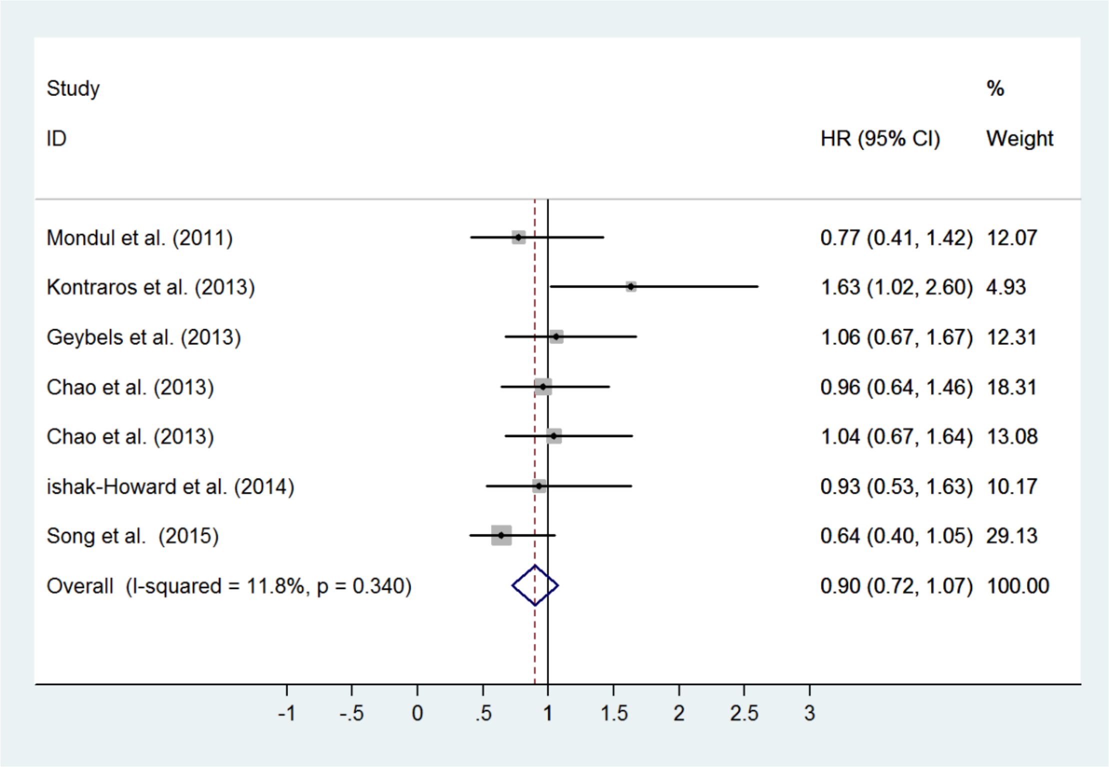

Additional file 4. Funnel plot for publication bias of 22 studies analysing BCR risk of prostate cancer.

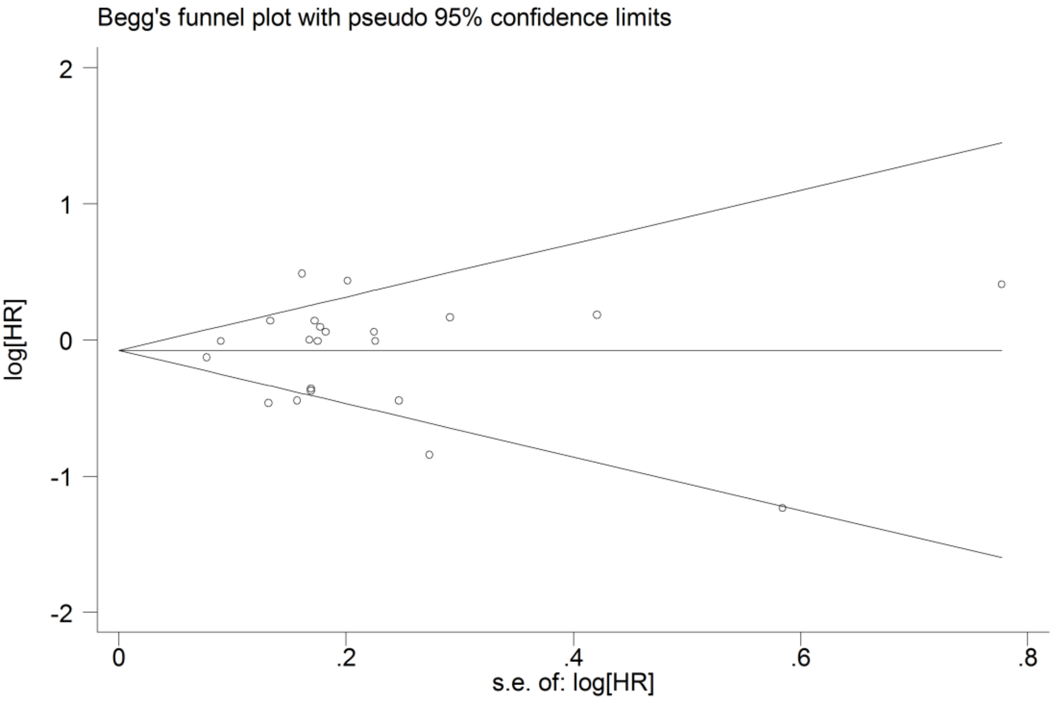

Supplement: Supplementary Information [file srep29106-s1.pdf]
